# Squamous cell carcinoma of the oral cavity – follow up treatment and distant metastatic behavior

**DOI:** 10.18632/oncoscience.582

**Published:** 2023-08-14

**Authors:** Florian Dudde, Ina Giersdorf, Filip Barbarewicz, Kai-Olaf Henkel

**Affiliations:** ^1^Department of Oral and Maxillofacial Surgery, Army Hospital Hamburg, Hamburg, Germany

**Keywords:** bone metastasis, squamous cell carcinoma, oral cavity, neck dissection, PET-CT

## INTRODUCTION

Up to 90% of tumors in the head and neck region are squamous cell carcinomas (SCC) [1]. SCC can develop in different anatomical sections of the head and neck. The SCC of the oral cavity is one of the most common locations [1].

The main risk factors for SCC in the oral cavity are chronic alcohol abuse and smoking [2]. Furthermore, chronic inflammation of the oral cavity and HPV infection predispose in exceptional cases to the development of SCC [1, 2].

SCC can manifest itself through various symptoms such as burning, pain and changes in the mucous membranes (leukoplakia, erythroplakia). A prosthesis that no longer fits as well as loose teeth can also indicate for SCC. However these symptoms may vary. Within the oral cavity, SCCs often occur in the area of the tongue, the floor of the mouth, as well as the jaw ridge and the palate [3].

Radiological imaging (computed tomography (CT) of the head, neck, thorax (abdomen) region and abdominal and cervical sonography, in special cases PET-CT) are linked to the tumor biopsy with subsequent histopathological confirmation of the findings [4]. The tumor size, the infiltration of neighboring structure and the presence/absence of pathological lymph nodes are essential for the further treatment planning [5].

The 5-year survival of isolated tongue carcinomas without evidence of lymphatic and hematogenous metastasis is >70% [1, 5]. Similarly high survival rates in the absence of metastasis can also be demonstrated for floor of the mouth carcinomas [1, 5]. In general, SCC of the head and neck region shows a primarily lymphatic metastatic behavior.

Hematogenous metastases (lungs, bones) in particular are rarely possible in advanced stages and/or recurrent SCC [6]. However, distant osseous metastases deriving from SCC of the head and neck region are to be considered a rarity, as we were able to clearly illustrate in a recent publication [7].

The therapy of SCC of the oral cavity consists of different pillars. Therapy usually begins with surgical removal of the tumor and, in particular because of the lymphatic metastasis behavior, is supplemented by selective neck dissection [1, 4]. In the case of positive lymph node involvement in the sense of a lymphatic-cervical metastasis, the neck dissection may be extended towards two stages (cervical lymph nodes neck dissection level IV and V) [1, 8]. Another pillar in the treatment of these tumors is (neo) adjuvant radiotherapy with/without chemotherapy, particularly in the case of extensive carcinomas including metastases [1, 9]. In some cases radiotherapy can also be indicated as a single therapy pillar. Additional challenges in the context of surgical therapy consist in the plastic defect coverage of tumor removal using various local and/or microsurgical distant flaps [10].

SCC recurrences occur primarily within the first two postoperative years [11]. Consequently, short clinical and radiological tumor follow-up is required in this regard. There should be 3-month follow-up intervals in the first two postoperative years and 6-month follow-up intervals in the 3rd to 5th postoperative year [1, 12].

Here, the follow-up care focuses in particular on the careful examination of the oral cavity and the throat to identify tumor recurrences in time. A retrospective study impressively showed that only 60% of all recurrent SCC of the oral cavity were symptomatic [13]. In particular, this underlines the need of a targeted clinical examination and the supplementation of radiological imaging procedures (CT/MRI of the head and neck). In addition to local recurrences, the occurrence of locoregional lymph node metastases is also associated with a reduced survival function in the head and neck area [1, 2].

However, depending on the initial tumor stage and the size of the recurrence, (re)operative therapy is a crucial pillar. In rare cases, SCCs in the oral cavity also tend to spread distant hematogenous metastases [1, 6]. As a rule, these are mainly hematogenous lung metastases. However osseous metastases, for example in the spine or bones of the upper extremities, are also possible [6].

In a recently published case report, we were able to associate a symptomatic distant osseous metastasis in the area of the femur with late recurrent SCC in the oral cavity [7]. Even if the described metastasis in the area of the femur must be regarded as an absolute rarity, this underlines the need for close and, above all, long-term tumor follow-up care. Furthermore, atypical complaints and symptoms outside the head and neck region can indicate (distant) metastasis and should therefore be clarified against an interdisciplinary background (clinic, radiology).
